# Tissue Doppler echocardiography and biventricular pacing in heart failure: Patient selection, procedural guidance, follow-up, quantification of success

**DOI:** 10.1186/1476-7120-2-17

**Published:** 2004-09-15

**Authors:** Fabian Knebel, Rona Katharina Reibis, Hans-Jürgen Bondke, Joachim Witte, Torsten Walde, Stephan Eddicks, Gert Baumann, Adrian Constantin Borges

**Affiliations:** 1 Charité Campus Mitte – University Medicine Berlin, Medical Clinic for Cardiology, Angiology, Pneumology, 10098 Berlin, Germany; 2 Klinik am See, Department of Cardiology, Rehabilitation Center of Cardiovascular Diseases, Seebad 84, 15562 Rüdersdorf (Berlin), Germany

**Keywords:** Echocardiography, Biventricular pacing, pacemaker programming, patient selection

## Abstract

Asynchronous myocardial contraction in heart failure is associated with poor prognosis. Resynchronization can be achieved by biventricular pacing (BVP), which leads to clinical improvement and reverse remodeling. However, there is a substantial subset of patients with wide QRS complexes in the electrocardiogram that does not improve despite BVP. QRS width does not predict benefit of BVP and only correlates weakly with echocardiographically determined myocardial asynchrony. Determination of asynchrony by Tissue Doppler echocardiography seems to be the best predictor for improvement after BVP, although no consensus on the optimal method to assess asynchrony has been achieved yet. Our own preliminary results show the usefulness of Tissue Doppler Imaging and Tissue Synchronization Imaging to document acute and sustained improvement after BVP. To date, all studies evaluating Tissue Doppler in BVP were performed retrospectively and no prospective studies with patient selection for BVP according to echocardiographic criteria of asynchrony were published yet. We believe that these new echocardiographic tools will help to prospectively select patients for BVP, help to guide implantation and to optimize device programming.

## Background

Heart failure is among the most common chronic diseases in modern civilizations. The dilatation of the left ventricle frequently induces intracardiac conduction delays resulting in asynchronous left ventricular motion. This manifests as left bundle branch block in the surface ECG. Both QRS width and intraventricular asynchrony are predictors of hospitalization and severe cardiac events in patients with heart failure [1-3].

The mechanisms of myocardial asynchrony include a delayed left ventricular regional contraction and relaxation. The right ventricle contracts during left ventricular end-diastole, leading to a "bulging" of the septum into the left ventricle. The intra(left)ventricular delay of the systolic velocity induces the "delayed longitudinal contraction (DLC)". Furthermore, the delay of the contraction of the papillary muscles aggravates mitral regurgitation. This, in summary, leads to an increased oxygen demand of the myocardium [4].

Resynchronization of the intraventricular conduction can be achieved by introducing an additional lead through the coronary venous sinus to stimulate the left ventricle (biventricular pacing, BVP). The combination of BVP and a cardioverter-defibrillator (ICD) combines the clinical improvement by BVP and reduction in mortality [5]. Recent studies have shown an acute and sustained hemodynamic improvement, reversal of LV-remodeling, an increased quality of life, a reduction of symptoms of heart failure, and improvement of exercise tolerance after biventricular pacing. Markers of reverse remodeling were reduction of left ventricular volumes, increase in LVEF without an increase in oxygen consumption, reduction of mitral regurgitation [6-10]. However, a significant reduction of mortality after BVP alone could not be demonstrated.

In the current guidelines, LBBB in the surface ECG and a reduced LVEF are the main indications for BVP [11]. However, about one third of patients in the large multicenter BVP studies did not improve – despite BVP [6,7,12]. There is increasing evidence, that there is only a weak correlation of electrical (QRS width) and mechanical asynchrony and the benefit of BVP. It seems that not all heart failure patients with LBBB have mechanical asynchrony [12,13].

Furthermore, asynchrony is common even in heart failure patients with narrow QRS complexes compared with healthy controls. A prospective study assessed left ventricular systolic and diastolic asynchrony in 67 patients with heart failure (LVEF < 50%) with normal QRS width and 45 patients with CHF and wide QRS complexes (>120 ms). 88 healthy control patients were included. Systolic (diastolic) asynchrony occurs in 51% (46%) of the heart failure patients with narrow QRS complexes and in 73% (69%) in the patients with wide QRS complexes. Systolic asynchrony was defined as the max difference in time-to-peak myocardial contraction of 12 myocardial segments. Diastolic asynchrony was defined as maximum difference of time-to-peak early diastolic relaxation. In summary, the authors state that asynchrony is common in patients with heart failure even without a wide QRS complex [14]. This is confirmed in a study with 158 heart failure patients (LVEF < 35%), that were divided in three subgroups: Group 1 with no (QRS < 120 ms), group 2 with mild (120–150 ms) and group 3 with severe LBBB (>150 ms). Interventricular asynchrony was defined by TDI as IVMD >40 ms and the intraventricular delay as maximum pre-ejection period of >50 ms in one or more myocardial segments. Asynchrony was seen in all three subgroups, however, there was no correlation between interventricular and intraventricular asynchrony [15].

A recent study demonstrated that successful BVP can be achieved in patients with a normal QRS duration and asynchrony [16,17].

These controversial data indicate the need for a more careful patient selection for BVP. Newer echocardiographic techniques, such as Tissue Doppler Imaging and Tissue Synchronization Imaging could potentially improve patient selection and guidance of implantation and programming of the devices for BVP. The risks of pacemaker implantation and expenses in non-responders to BVP could be avoided. Furthermore, the cost-effectiveness of BVP would be augmented.

## Definitions of asynchrony

Regarding the nomenclature, the term "asynchrony" is used synonymously to "dyssynchrony" in this article. There is a variety of methods to determine asynchrony. In table 1, the different approaches to asynchrony are listed concisely. The QRS width (LBBB > 120, 130, 150 ms) is the simplest method, but the sensitivity to predict benefit from BVP is rather low [18,19]. Magnetic resonance imaging can also detect areas of asynchrony but this technique can not be repeated for follow-up after device implantation.

**Table 1:** Concise summary of the different approaches to echocardiographic measurement of asynchrony

**Assessment of asynchrony with:**	**Ref.**	**Criteria**	**Segments**	**Limitations**	**Analysis time**	**Prediction of benefit**
**I. Global ventricular asynchrony**						

**ECG**	4, 44	QRS width >120 ms	Global assessment	LBBB after myocardial infarction	Short	Low (30% non-responder)
**M-mode**	21	Septal-to-posterior wall motion delay >130 ms	septal and posterior	scar tissue, only septal or posterior	Short	low
**pw-TDI**	25	Cumulative asynchrony (EMD) >102 ms	Intra LV (5 basal segments) and interventricular (vs. RV lateral segment)	Low spatial resolution	Long	Good prediction of acute response (AUC in ROC 0,84)

**II. Interventricular asynchrony**						

**pw-Doppler echocardiography**	47	Interventricular mechanical delay (IVMD) >40 ms	Aortic and pulmonary outflow tract	Not simultaneous	Short	No

**III. Intraventricular asynchrony**						

**2D-TDI**	27	Ts-SD: intraventricular systolic asynchrony index: >33 ms	12 segments	complex (post-processing)	Long	Acute response (3 months)
	4, 32	Difference in septal-lateral time-to-peak TDI, cut-off >60 ms	12 segments	Complex	Long	EF increase after BVP
	40	Mean regional myocardial performance index: Difference between regional Q-wave-to.peak systolic displacement times	12 segments 4 segments	Complex	Long, offline	Acute response
	33	Ts-SD: cut-off: 31,4 ms	12 segments	Complex	Long	3 months response, reverse remodeling
**Strain and strain rate**	15, 34, 33	Myocardial deformation in systole, presence of post-systolic shortening	12 segments	Complex, time consuming, in dilated ventricles low spatial resolution)	Long	Controversial data
**Tissue Tracking**	28	DLC in >2 basal segments	12 basal segments in apical four chamber view.	Requires correct timing of LV events	Short	Acute response
**TSI**	36	Color-coded time-to-peak tissue Doppler velocities (cut-off >65 ms in anteroseptum and posterior wall in apical long axis view)	16 segments except apex	Only velocity data	Short	Acute response (Sensitivity 87% Specificity 100%)
**3-D-echo**	26	No quantitative criteria defined	All segments	Reduced spatial resolution	Time consuming, off-line analysis	No systematic data
**Automated endocardial border detection (ABD)**	26	Septal-lateral phase angle difference	100 segments. apical-four-chamber view (septal-lateral)	High complexity, single imaging plane	Long, only off-line	Acute response
**ABD + Contrast**	46	Echo-contrast cardiac variability imaging: displacement maps	apical four chamber	High complexity, single imaging plane	Long	Acute response

Echocardiographic tools include 2D, Doppler and Tissue Doppler methods. Up to date, there is no consensus on the definition of echocardiographically measured myocardial asynchrony. The determination of asynchrony by M-mode echocardiography is limited to septal and inferior segments in parasternal long-axis and is not performed routinely in current studies [20,21]. Earlier echocardiographic approaches to asynchrony included the delayed long axis shortening that was found to suppress early diastolic transmitral flow and subsequently leading to decreased leftventricular function [22].

Tissue Doppler imaging (TDI) measures regional wall motion velocities. TDI can accurately quantify regional left ventricular function [23]. Pulsed wave TDI does not allow simultaneous comparison of regional timing in different segments within one cardiac cycle. Color-coded TDI reduces beat-to-beat variability and examination time. Color coded TDI has a very high time resolution of 10 ms.

TDI technology includes tissue tracking and strain rate imaging. Tissue tracking allows the measurement and visualization of longitudinal motion in each myocardial segment during the different phases of the heart cycle.

Strain measures compression and distension of myocardial segments ("deformation imaging") and strain rate imaging expresses strain changes per time interval. Post-systolic movement diagnosed with velocity or tissue tracking can be differentiated into passive or active motion (=contraction, then defined as PSS). But in ischemic cardiomyopathy PSS was not an useful criterion for response to BVP because this phenomenon is not only a sign of asynchrony but also a marker for ischemia and/or viability of severe hypo/akinetic segments [24].

Tissue synchronization imaging (TSI) is a newer technique that utilizes color-coded time-to-peak tissue Doppler velocities and visualizes segments of dyssynchrony in real-time by superimposing these temporal motion data on 2D echo images. TSI analysis is possible in all myocardial regions except the apex. The color-coding is green (normal time-to-peak velocity: 20–150 ms), yellow (150–300 ms) and red (300–500 ms) [25]. Online 3D echocardiography and automated border detection (ABD) might be future diagnostic tools to diagnose asynchrony but need evaluation in larger studies [26].

Myocardial asynchrony includes inter- and intraventricular asynchrony. Interventricular asynchrony can be assessed by comparing pw-Doppler signals in the right and left ventricular outflow tracts. A delay of >60 ms is considered to demonstrate interventricular asynchrony. These measurements in the outflow tracts cannot be performed simultaneously and, therefore, there is a high inter-measurement variability and dependence on cardiac workload. In addition, interventricular asynchrony can measured as the difference of the electromechanical delays in the basal LV segments and the lateral RV segments [35].

Intra(left)ventricular asynchrony is considered to be the most important aspect of the electromechanical delay (EMD). It can be measured by a variety of methods. EMD is defined as the delay between the onset of the QRS complex on the surface ECG and the onset of the systolic TDI wave in corresponding myocardial segments. Recently, the systolic synchronicity index has been introduced [27]. It is defined as the standard deviation (SD) of the EMD in 12 LV segments (6 basal, 6 mid-segmental model).

Intraventricular asynchrony can also be demonstrated by tissue tracking with diastolic color-coded areas called DLC. This is the amount of post-systolic contraction after the closure of the aortic valve (i.e. post systolic shortening = PSS) which was confirmed by strain and strain rate in this study [28].

Intra-left ventricular asynchrony is not only of diagnostic value for selecting patients for BVP, but has prognostic value as well. Bader et al. [3] examined inter- and intraventricular asynchrony as an independent predictor of heart failure worsening: 104 patients with chronic stable heart failure without previous myocardial infarction (LVEF < 45%) were included, follow-up echocardiography was performed after one year. Study endpoint of heart failure worsening was hospitalization for cardiac decompensation. Intra-left ventricular asynchrony is an independent predictor of severe cardiac events. Only a weak correlation of intra/inter-ventricular EMD and QRS width could be demonstrated.

In figures 1, 2, 3, 4, 5, 6, 7, 8, 9, the different approaches to assess asynchrony as well as echocardiographic examples of successful biventricular pacing are illustrated.

**Figure 1:**
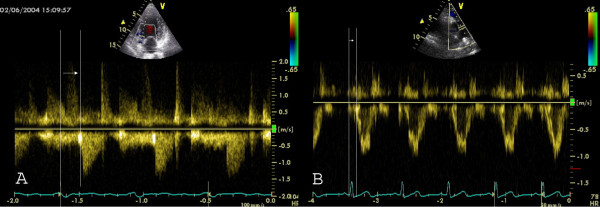
Measurement of **interventricular mechanical (IMD) delay by PW Doppler**: A) PW Doppler in aortic outflow tract: Measurement from onset of QRS to the onset of PW curve in the aortic outflow tract. This time is also called the aortic pre-ejection time and is a marker for intra(left)ventricular asynchrony. B) PW Doppler in pulmonary outflow tract: Measurement from onset of QRS to the onset of PW curve in the pulmonary outflow tract. The IMD is the difference between the time of a) and b).

**Figure 2:**
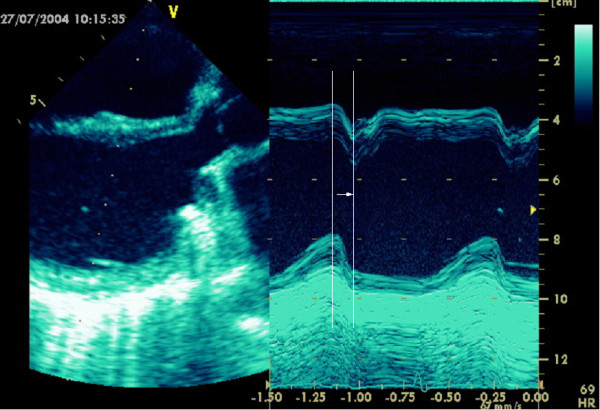
Assessment of asynchrony in parasternal long axis view by **M-mode**: Time difference between peak of septal and inferior myocardial contraction.

**Figure 3:**
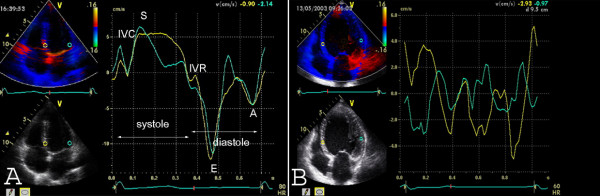
**Tissue Doppler velocity **data for the quantification of asynchrony from apical four chamber view. Sample volumes are in the basal lateral and basal septal segment. A) Normal control patient. There is a synchronous myocardial velocity in the septal (=yellow) and the lateral (=green curve) segment. IVC = isovolumetric contraction, IVR = isovolumetric relaxation, S = peak systolic velocity; E = early diastolic filling, A = late (atrial) diastolic filling. B) There is asynchronous myocardial velocity in the septal (=yellow) and the lateral (=green curve) segment.

**Figure 4:**
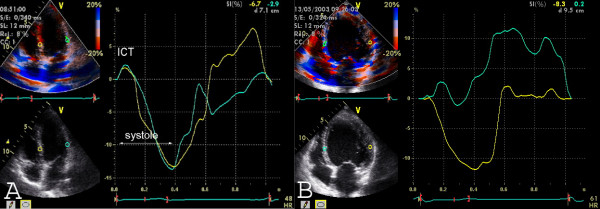
Assessment of asynchrony by **strain **from the apical four chamber view. The sample volumes are in the basal septal and the basal lateral segments. A) Normal strain curve in a control patient. ICT = isovolumetric contraction time. B) Strain curve with asynchronous myocardial velocity in the septal (=yellow) and the lateral (=green curve) segment.

**Figure 5:**
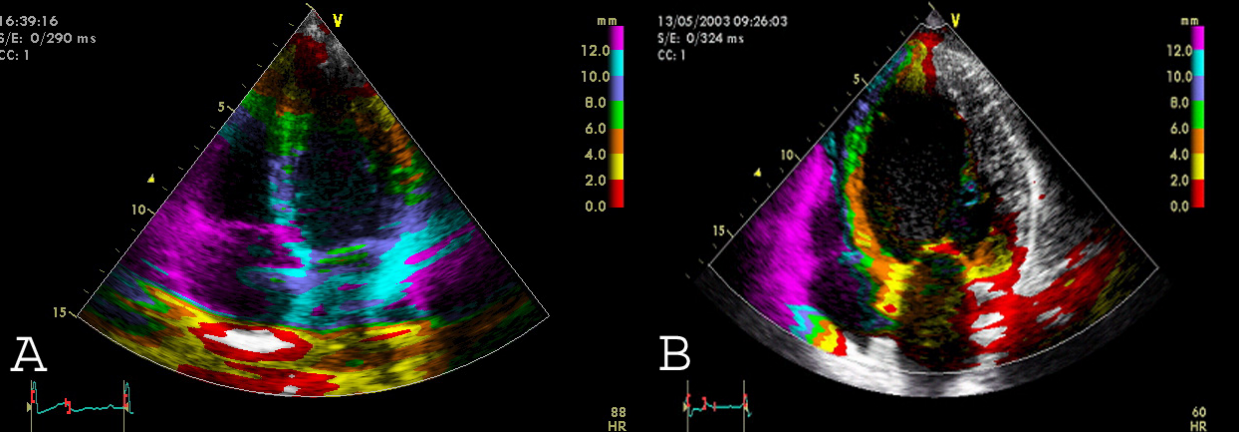
**Tissue Tracking **allows the visualization of longitudinal motion in each myocardial segment. Images are from the apical four chamber view. A) Normal control patient. There are normal colour-coded displacement values in the lateral and septal segments, with physiologically higher values in the more basal segments and lower values towards the apex. B) Tissue Tracking in a patient with dilated cardiomyopathy. There is a dilated left ventricle with "baseball shape" and reduced displacement values and no basal-apical gradient (max displacement = 8 mm) in the septal segments and DLC in the lateral wall (no colour-coding) indicating asynchrony of the lateral wall.

**Figure 6:**
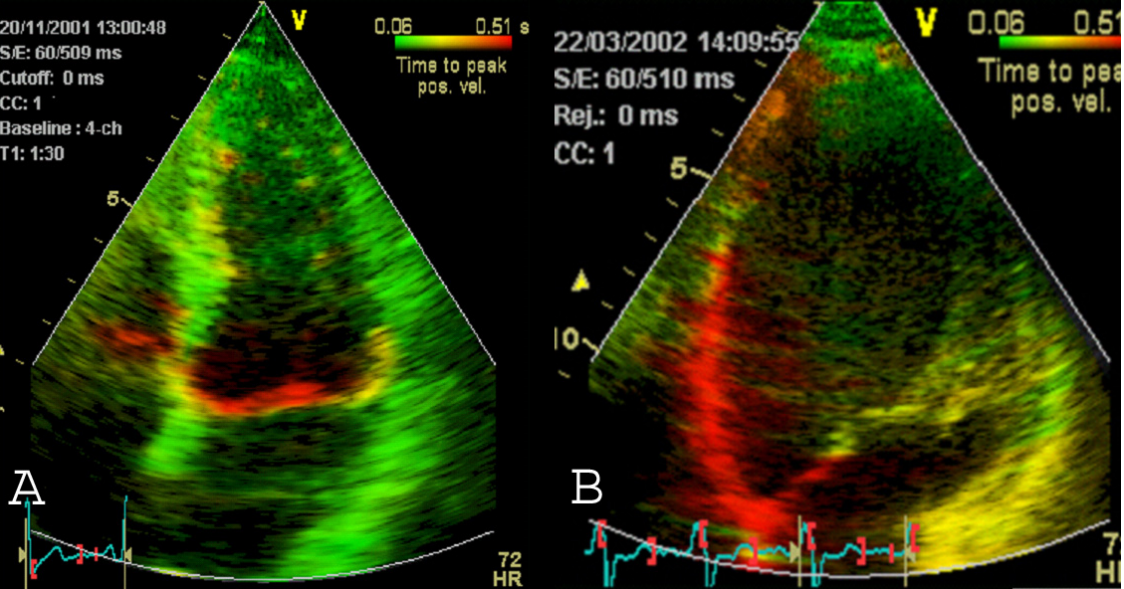
**Tissue Synchronization Imaging **displays colour-coded time-to-peak tissue Doppler velocities. The colour-coding is green (normal time-to-peak velocity: 20–150 ms), yellow (150–300 ms) and red (300–500 ms) Apical four chamber view. A) TSI in a control patient (only green colour coding indicating synchronous contraction) B) TSI in a patient with LBBB: The basal and mid-septal segments show a delayed time-to-peak velocity (red colour).

**Figure 7:**
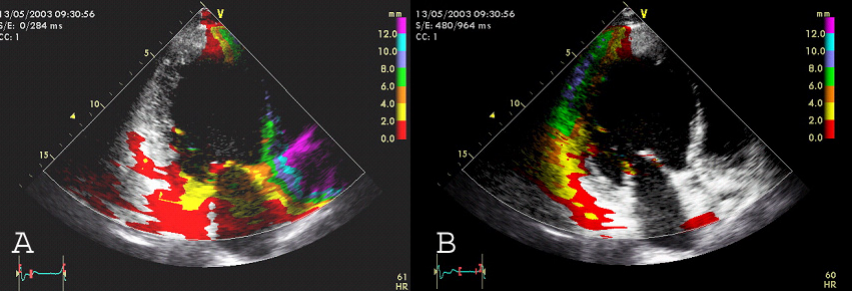
Delayed longitudinal contraction (=DLC) as a marker for asynchrony can be visualized by analysis of **systolic and diastolic Tissue Tracking**. Systolic tracking analyzes the systolic displacement i.e. tracking interval between the onset of QRS-complex and the end of the T-wave. Diastolic tracking can demonstrate DLC with colour coding (end of T until begin of R). Images from apical two chamber view A) Systolic Tracking: The inferior segments (=grey area) show DLC with no systolic motion B) Diastolic Tracking: The inferior segments (=colour coded area) show DLC with diastolic movement.

**Figure 8:**
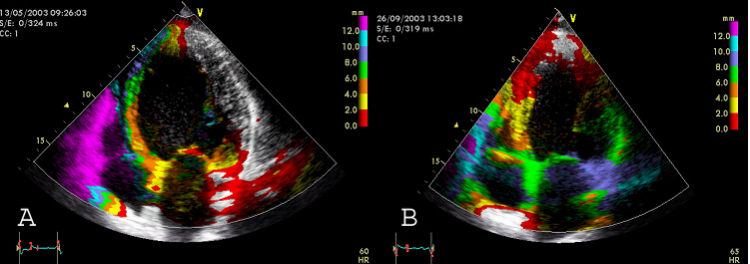
Demonstration of successful BVP by Tissue Tracking in apical four chamber view in a patient with dilated cardiomyopathy. Images from apical four chamber view. A) Before BVP, there is a dilated ventricle ("baseball shape") with reduced systolic displacement (max displacement = 8 mm) in the septum and DLC in the lateral wall (no colour-coding) indicating asynchrony of the lateral wall. B) After three months of BVP, there is a reduction of left ventricular dilatation (reverse remodelling, "American football shape" of the left ventricle), increased tracking values and no DLC regions anymore.

**Figure 9:**
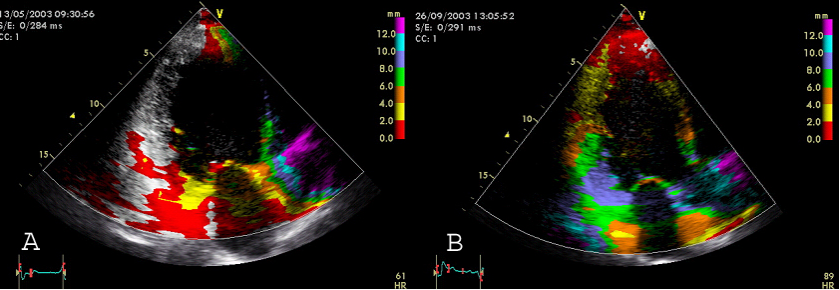
Successful BVP documented by Tissue Tracking in apical two chamber view. A) Before BVP, there is a dilated ventricle with reduced systolic displacement (max displacement = 8 mm) in the septum and DLC in the inferior wall (no colour-coding) B) After three months of BVP, there is a reduction of left ventricular dilatation (reverse remodelling), increased tracking values, a basal-apical gradient and no DLC regions anymore.

## Patient selection for BVP

Only limited data are published concerning prospective echocardiography based patient selection for BVP. Bordachar et al. [29] performed a prospective study to identify TDI parameters that would predict the benefit of upgrading right ventricular pacing to BVP. 26 patients with normal LVEF and RVP and 16 patients with CHF and RVP were included. EMD was defined as the interval between the stimulation spike and the onset of the S wave. An intra-ventricular EMD of >50 ms identifies patients with significant asynchrony. No correlation between asynchrony and QRS width was seen in the heart failure patients. ECG criteria would have misclassified 44% of the patients for mechanical ventricular asynchrony. This study has defined relevant asynchrony but did not assess the hemodynamic or electromechanical effects after upgrade to BVP nor effects on morbidity and mortality.

## Retrospective analysis after BVP

Several studies were performed to retrospectively correlate markers of asynchrony to benefit from BVP.

Lafitte [30] has included 15 patients with idiopathic DCM and a QRS of more than 140 ms (NYHA III-IV, LVEF < 35%, LVEDD > 60 mm) for BVP. Measurement of EMD was performed at baseline and after one month. This study has found that BVP reduces EMD in the lateral left ventricular wall.

In another study [25], 49 patients with heart failure (QRS > 130 ms, LVEF < 35%, NYHA II-IV) were included. Retrospectively, intra- and interventricular and the combined index of asynchrony (=the sum of left and right ventricular asynchrony) were assessed at baseline and after 6 months of BVP by pulsed wave TDI. The cut-off-values for LV-asynchrony was 60 ms (56 ms for RV-LV-asynchrony and 102 ms for the "sum-asynchrony"). By definition, patients with a relative increase in LVEF of more than 25% were classified as responders to BVP. Receiver-operating characteristics (ROC) analysis showed that the degree of echocardiographic asynchrony is superior to QRS width in predicting hemodynamic and clinical improvement after BVP compared to QRS duration or conventional echo data. In 82% of the patients, the benefit of BVP could have been predicted echocardiographically.

The role of TDI and 3D echo on the long term (1 year) outcome after BVP was evaluated in 25 patients [19]. The extent of DLC in the basal segments at baseline predicted the long-term efficacy of BVP. The LV base DLC was reduced from 18,7% to 8.1% after BVP. In concordance with other studies, the QRS duration failed to predict BVP efficacy [28].

The myocardial segments with the best resynchronization after BVP were studied in 18 patients with an LVEF <35% and a QRS width of >120 ms (NYHA III-IV). Color tissue Doppler velocity imaging was performed from the apical four chamber view at baseline and one month of follow-up after BVP [31]. Peak velocities and regional time differences in basal and mid septal segments were compared to the corresponding lateral segments. At baseline, a regional asynchrony of 42 ms in the basal sites (only 14 ms in the mid left ventricular site) was measured. After one month of BVP, a reduction of asynchrony was seen in only the basal segments but not in the mid segments. In conclusion, it was suggested that hemodynamic improvement is mainly in basal sites.

Reverse remodeling and improved synchrony after 3 months of BVP was evaluated in 25 patients [32]. Asynchrony was assessed as time-to-peak regional sustained systolic contraction (=Ts). After three months, a homogenous left ventricular delay of Ts, improved interventricular synchrony and a reduced isovolumic contraction time and increased diastolic filling time were documented. These beneficial effects were reversible after withholding BVP. In a univariate analysis, systolic dyssynchrony was the only independent predictor of reverse remodeling after three months [33].

One recent study has compared the value of TDI and SRI and post-systolic shortening in the prediction of reverse remodeling after BVP: The previously introduced asynchrony index (=Ts-SD) based on Tissue Doppler velocity data has the highest predictive value of reverse remodeling after BVP. PSS has predictive power only in non-ischemic heart failure. In ischemic heart failure, PSS seems not to be a marker for reverse remodeling but rather reflects viability and is therefore not altered by BVP. SRI imaging techniques did not predict reverse remodeling after three months of BVP [33]. This is in contrast to previously published data [34].

Kanzaki has introduced the synchrony index, which is defined as the correlation coefficient of linear regression of velocity of septal and lateral mitral annular region. This index showed an increase after 6 months of BVP paralleled by increased LV contractility [35].

One study [36] has retrospectively evaluated the use of TSI to predict the acute response to BVP in 29 patients. The acute benefit to BVP was defined as a >15% increase in echocardiographically measured stroke volume 48 h after device implantation. A difference of >65 ms in time-to-peak velocity in anteroseptal and posterior segments in the apical long axis view was associated with acute improvement after BVP. However, the ability of TSI to predict long-term improvement after BVP needs further evaluation.

## Guidance for implantation

TDI could play a role in identifying patients during catheterization procedures that will profit from BVP. Catheterization studies have shown that the beneficial effects of BVP begin almost immediately [37,38]. But systematic evaluation with TDI-technique is currently ongoing.

Furthermore, TDI can assist in finding the optimal pacing site for the coronary sinus lead. In 31 patients, it was documented that LV-stimulation on the site of longest EMD had the best benefit of BVP. The regional asynchrony was assessed by pw-TDI and the pacing site was determined fluoroscopically [39]. Lateral and postero-lateral LV lead positions were retrospectively found to improve left ventricular hemodynamics [40].

## Optimal programming of biventricular device after implantation

### AV-time programming

An AV time is considered to be optimal when the end of the A wave coincides with the complete closure of the mitral valve [41]. An optimal AV time setting of the pacemaker can improve systolic function [42]. However, there is only limited published data assessing the optimal AV time in patients with BVP.

### Optimization of the interventricular delay

The optimal delay between the right ventricular and the coronary sinus stimulation is yet unknown. One study compared simultaneous versus sequential BVP in 29 patients. The optimum interventricular delay was found by maximum reduction of DLC as measured by Tissue Doppler and Tissue Tracking. An optimum sequential BVP could significantly reduce the extent of DLC compared to simultaneous pacing [43].

## Patients with atrial fibrillation

About one third of patients with heart failure have atrial fibrillation. The large trials, however, have only included patients in sinus rhythm. Only small studies with controversial results were performed in patients with atrial fibrillation and LBBB. Leclercq has performed one study in 59 NYHA III patients with chronic atrial fibrillation, a slow ventricular rate and the need for permanent pacing (VVI-paced QRS width of >200 ms). Due to a high drop out rate, the results did not show a significant increase in 6-min-walk distance after BVP [44]. Larger trials are needed to evaluate BVP for patients with atrial fibrillation.

## Preliminary own results

We have performed a double-blind cross-over study in our clinic to assess the use of new echocardiographic techniques in BVP. Patients (n = 40) with a QRS >140 ms and a LVEF <35% received an InSyncICD 7272 (Medtronic, Minneapolis, Minnesota, USA). Preliminary results (n = 8) after two years demonstrate a reduction of the septal-posterior delay from 264 (±23) msec to 234 (±34) msec (p < 0,05) and a stabilization of clinical (NYHA class improvement) and hemodynamic status (EF and LV volumes). The study is ongoing.

The following video loops underline the utility of TSI and Tissue Tracking to document improvement of synchronicity after BVP. In additional file 1 shows asynchrony before BVP implantation in apical four chamber view by TSI. In additional file 2 the effect of BVP is shown. in additional file 4 shows the acute changes of BVP as documented in this video loop by Tissue Tracking from apical four chamber view compared to baseline (additional file 3). The long-term effect of BVP after six months is illustrated in additional file 5 (baseline) and additional file 6 (after 6 months).

## Conclusion and future perspective

Many controlled and uncontrolled studies have demonstrated that new echocardiographic tools to determine myocardial asynchrony in heart failure patients will help to select patients for BVP help guidance of implantation and optimize device programming. To date, all studies employing tissue Doppler date were performed retrospectively. No prospective studies that have selected patients for BVP according to echocardiographic evaluation of asynchrony were performed yet. The ongoing CARE-HF study incorporates echocardiographic criteria of asynchrony in a subset of patients with a QRS of 120–150 ms [45]; results are not expected until 2005. The criteria of asynchrony in this study are (1) aortic pre-ejection delay >140 ms, (2) the mechanical interventricular (pw aortic valve vs. pulmonary valve) delay >40 ms and (3) the demonstration of left ventricular post-systolic contraction by M-mode and/or Tissue Doppler.

Unresolved issues include different opinions regarding the various elements of asynchrony and their contribution to the pathophysiology and progression of heart failure. There is a lack of consensus about the best asynchrony marker for patient selection. There is evidence that ischemic and dilated cardiomyopathy might have different selection parameters for BVP. The practical consequences for patient selection and/or implantation site of the lead are currently under investigation. There are only limited echocardiographic data regarding the programming of the optimal interventricular (V-V) delay. There are no data concerning the long-term effect (i.e. years) of BVP on hemodynamics, amelioration of mitral regurgitation, reverse remodeling and mortality. Another area of uncertainty is the selection of patients for BVP without electrical (QRS < 120 ms) but with mechanical asynchrony.

## Abbreviations

BVP Biventricular Pacing

DCM Dilated Cardiomyopathy

DLC Delayed longitudinal Contraction

EMD Electromechanical Delay

IVMD Interventricular Mechanical Delay

LBBB Left Bundle Branch Block

PSS Post-Systolic Shortening

SRI Strain Rate Imaging

TDI Tissue Doppler Imaging

Ts Time-to-peak myocardial contraction

TSI Tissue Synchronization Imaging

Ts-SD Standard deviation of time-to-peak myocardial contraction

## Authors contributions

F Knebel and AC Borges have performed the literature review and have prepared the manuscript. RK Reibis, have performed echocardiographic examinations for this article. HJ Bondke and J Witte and G Baumann have selected patients for BVP. HJ Bondke and J Witte have implanted the biventricular pacing devices. All authors have read and approved the final version of the manuscript.

## Supplementary Material

Additional File 1 TSI in a patient with LBBB before BVP: The lateral segments show a delayed time-to-peak velocity (red colour). Apical four chamber view. Click here for file

Additional File 2 TSI post-implantation: There is only green colour coding indicating synchronous contraction of all segments from apical four chamber view. Click here for file

Additional File 3 Tissue Tracking without BVP : There are reduced displacement values and no basal-apical gradient in the septal segments and DLC in the lateral wall (no colour-coding) indicating asynchrony of the lateral wall. Apical four chamber view. Click here for file

Additional File 4 Acute effect with BVP "on" (Tissue Tracking): There are increased displacement values, a basal-apical gradient. Apical four chamber view. Click here for file

Additional File 5 Tissue Tracking before BVP : There are reduced displacement values and no basal-apical gradient in the septal segments and DLC in the lateral wall. Apical four chamber view. Click here for file

Additional File 6 Long-term effect after 6 months of BVP: Reduction of left ventricular dilatation, increased displacement values, a basal-apical gradient. Apical four chamber view. Click here for file
